# Circulating Mediators of Inflammation and Immune Activation in AIDS-Related Non-Hodgkin Lymphoma

**DOI:** 10.1371/journal.pone.0099144

**Published:** 2014-06-12

**Authors:** Brian M. Nolen, Elizabeth Crabb Breen, Jay H. Bream, Frank J. Jenkins, Lawrence A. Kingsley, Charles R. Rinaldo, Anna E. Lokshin

**Affiliations:** 1 University of Pittsburgh Cancer Institute, University of Pittsburgh, Pittsburgh, Pennsylvania, United States of America; 2 Department of Psychiatry & Biobehavioral Sciences, David Geffen School of Medicine at UCLA, University of California Los Angeles, Los Angeles, California, United States of America; 3 UCLA AIDS Institute, University of California Los Angeles, Los Angeles, California, United States of America; 4 Department of Molecular Microbiology and Immunology, Johns Hopkins Bloomberg School of Public Health, Johns Hopkins University, Baltimore, Maryland, United States of America; 5 Department of Pathology, School of Medicine, University of Pittsburgh, Pittsburgh, Pennsylvania, United States of America; 6 Department of Infectious Diseases and Microbiology, Graduate School of Public Health, University of Pittsburgh, Pittsburgh, Pennsylvania, United States of America; 7 Department of Medicine, School of Medicine, University of Pittsburgh, Pittsburgh, Pennsylvania, United States of America; 8 Department of Ob/Gyn, School of Medicine, University of Pittsburgh, Pittsburgh, Pennsylvania, United States of America; New York University, United States of America

## Abstract

**Background:**

Non-Hodgkin lymphoma (NHL) is the most common AIDS-related malignancy in developed countries. An elevated risk of developing NHL persists among HIV-infected individuals in comparison to the general population despite the advent of effective antiretroviral therapy. The mechanisms underlying the development of AIDS-related NHL (A-NHL) are not fully understood, but likely involve persistent B-cell activation and inflammation.

**Methods:**

This was a nested case-control study within the ongoing prospective Multicenter AIDS Cohort Study (MACS). Cases included 47 HIV-positive male subjects diagnosed with high-grade B-cell NHL. Controls were matched to each case from among participating HIV-positive males who did not develop any malignancy. Matching criteria included time HIV+ or since AIDS diagnosis, age, race and CD4+ cell count. Sera were tested for 161 serum biomarkers using multiplexed bead-based immunoassays.

**Results:**

A subset of 17 biomarkers, including cytokines, chemokines, acute phase proteins, tissue remodeling agents and bone metabolic mediators was identified to be significantly altered in A-NHL cases in comparison to controls. Many of the biomarkers included in this subset were positively correlated with HIV viral load. A pathway analysis of our results revealed an extensive network of interactions between current and previously identified biomarkers.

**Conclusions:**

These findings support the current hypothesis that A-NHL develops in the context of persistent immune stimulation and inflammation. Further analysis of the biomarkers identified in this report should enhance our ability to diagnose, monitor and treat this disease.

## Introduction

Non-Hodgkin lymphoma (NHL) is the most common AIDS-related malignancy in developed countries [1], where it accounts for 23–30% of AIDS-related death [2]–[4]. Although NHL also affects HIV-uninfected individuals, the risk of developing AIDS-associated NHL (A-NHL) is estimated to be 60 times greater in HIV-infected (HIV+) persons [5], [6]. A recent study regarding the incidence of AIDS-related cancer in the years prior to and during widespread administration of highly active antiretroviral therapy (HAART) indicated that while HAART implementation is associated with a reduction in NHL rates among HIV+ persons, rates of A-NHL development remain significantly higher than those observed in uninfected populations [7]. Immunosuppression of any type appears to be a major risk factor for NHL development, with duration of HIV infection and nadir CD4-positive (CD4+) T cell counts positively and inversely associated with the risk of A-NHL, respectively [8], [9]. The incidence of primary central nervous system lymphoma (PCNSL), a less prevalent subtype of NHL associated with immune deficiency, has been reported to be over 1000 times higher in persons with AIDS compared to the general population [10], [11], although recent evidence suggests that this incidence is declining dramatically with the advent of HAART [12].

The precise pathogenic mechanism underlying A-NHL development is a current focus of intense scrutiny, and several basic themes have emerged. Immune deficiency appears to contribute to the development of A-NHL through the loss of T-cell mediated control over B-cell proliferation. This mechanism appears to be particularly important in the development of Epstein-Barr virus (EBV)-positive A-NHL (reviewed in [13]). The persistently elevated incidence of A-NHL despite the advent of HAART argues for additional pathogenic mechanisms including hyperactivation of B-cells. Chronic B-cell hyperactivation associated with genetic abnormalities due to EBV or human herpesvirus-8 (HHV-8) infection, c-MYC and BCL-6 rearrangements, RAS or p53 mutations, and 6 q deletions is believed to play a role in A-NHL [6], [14], [15]. Direct integration of HIV into the genome of malignant B-cells has not been observed experimentally, however persistent antigenic stimulation of B-cells during HIV infection may promote hyperactivation and transformation [16]–[19]. Another proposed mechanism suggests that HIV-infected macrophages contribute stimulatory signals in order to create a microenvironment permissive to malignant B-cell growth [20].

The proposed pathogenic pathways underlying the development of A-NHL suggest a complex network of interactions between various components of the immune system. Cytokines and other inflammatory mediators represent the chief means by which these components mediate those interactions. A number of groups have evaluated serum biomarkers in an effort to more fully characterize the dysregulated cytokine signalling underlying the immunological mechanisms at work in the development of A-NHL. A number of proteins involved in B-cell activation, stimulation and other inflammatory functions have been implicated by the findings of those groups including: IL-6 [21], [22], IL-10 [21]–[23], IP-10 [22], neopterin [22], immunoglobulin free light chains [22], sCD30 [21], [24], sCD23 [21], [25], sCD44 [26], CRP [21], CXCL13 [27], [28] and TNFα [22]. Many of these proteins were observed at elevated serum levels in subjects prior to the diagnosis of A-NHL with a lead time ranging from several months to several years [21], [23], [24], [26]. These findings suggest that the analysis of serum biomarker levels in HIV+ individuals may help in risk assessment strategies for NHL or in the development of prophylactic or preventive measures through an improved understanding of the early events of malignancy.

In the current study, we sought a broad evaluation of serum biomarkers that could possibly be associated with the development of NHL in HIV+ individuals. To that end, multiplexed bead-based immunoassays were utilized in the evaluation of serum levels of 161 proteins in subjects diagnosed with A-NHL and controls.

## Materials and Methods

### Ethics Statement

All subjects involved in this study were over the age of 18 and provided written informed consent. Specimen collection procedures were approved by the respective Institutional Review Board (IRB) at each collection site: Johns Hopkins Medicine IRB, Northwestern University IRB, University of California, Los Angeles IRB, University of Pittsburgh IRB. The current study was approved by the University of Pittsburgh IRB.

### Study Population

The Multicenter AIDS Cohort Study (MACS) is an ongoing prospective study of the natural and treated histories of HIV-1 infection in homosexual and bisexual men conducted at sites located in Baltimore, Chicago, Pittsburgh and Los Angeles [29]. A total of 6,972 men have been enrolled. For the current analysis we obtained archived sera from HIV+ subjects diagnosed with high grade B-cell NHL (according to the MACS data repository) for which serum was available either immediately prior to (≤12 months, n = 37) or shortly after (≤3 months, n = 10) NHL diagnosis. For each case, a single matched control was identified from among HIV+ participants who did not develop any malignancy. Cases for which NHL was AIDS-defining (n = 25) were matched to controls who had not yet received an AIDS diagnoses at the time of blood collection, based on duration of HIV+ status (+/−1 year). Cases for which NHL developed subsequent to an AIDS diagnosis (n = 22) were matched to controls who had developed an AIDS-defining opportunistic infection (OI), based on time since AIDS diagnosis (+/−1 year). Matching criteria also included age (+/−5 yrs) and absolute CD4+ T cell count (+/−100 cells/mm^3^) at the time of blood collection in the case or matched visit in the control, and race (white or black). Following matching, a total of 49 cases and 49 controls were selected. Sera obtained for three subjects (2 cases, 1 control) were not evaluable due to insufficient volume or hemolysis. In the resulting groups of 47 cases and 48 controls (Table 1), age, race distribution, CD4+ T cell counts, and duration of HIV positivity did not differ significantly (p>0.05), reflecting the original matching criteria. The final experimental cohort included 46 matched pairs. 46 of the cases included in the current study overlap with those used in recent studies utilizing the MACS cohort [21], [27], [30]. In the majority of overlapping cases, the date of serum collection for the current study was closer to or after the A-NHL diagnosis date, compared to previous studies, as determined by the specific selection criteria. Information on NHL subtypes was obtained from the MACS data repository. HIV viral load values obtained at time of blood draw for biomarker analysis were available from the MACS data repository for 36 case and 22 control subjects. Viral load values were not utilized for matching but were utilized in subsequent biomarker analyses. Information on antiretroviral therapy was obtained from the MACS data repository for each case and control. HAART regimens were defined according to the DHHS/Kaiser guidelines [31]. Subjects were classified as current recipients if they received HAART at the same visit as their blood draw or during the interim time in between this visit and the previous MACS visit. Subjects were classified as former recipients if they received HAART prior to but not during this time period. Subjects classified as never receiving HAART may have received other antiretroviral therapy not classified as HAART. The characteristics of the study population are presented in Table 1.

**Table 1:** Characteristics of Study Population.

	A-NHL Cases (n = 47)	Controls (n = 48)
Age (years), range (median)	25–56 (42)	26–56 (40)
Race, n (%)		
White	46 (98)	47 (98)
Black	1 (2)	1 (2)
CD4+ T Cell Count (cells/mm^3^), range (mean)	7–788 (146)	6–794 (193)
Receiving HAART [31], n (%)		
Current	6 (13)	5 (10)
Former	1 (2)	1 (2)
Never	38 (81)	42 (87.5)
Unknown	2 (4)	0 (0)
HBV Serology, n (%)		
Positive	41 (87)	42 (87.5)
Negative	3 (6)	6 (12.5)
Unknown	3 (6)	0 (0)
HHV-8 Serology, n (%)		
Positive	28 (57)	35 (73)
Negative	9 (19)	9 (19)
Unknown/Indiscriminate	10 (21)	4 (8)
NHL Subtype, n (%)		
B-cell Diffuse	20 (43)	
CNS	11 (23)	
BL, BL-like	7 (15)	
NHL, NOS	6 (13)	
Other	3 (6)	

HAART – highly active antiretroviral therapy; HBV/HHV-8 status obtained at time of blood draw; CNS – central nervous system; BL – Burkitt's lymphoma; NOS – not otherwise specified.

### Sources of bead-based immunoassays

A combination of 161 bead-based immunoassays for a diverse set of serum biomarkers available on the Luminex platform was utilized in this study (Table 2). This list of biomarkers was assembled based on a literature review of proteins and families of proteins of interest in all areas of HIV and NHL research. Biomarkers were selected from this list on the basis of suitable immunoassay availability. A total of 29 multiplexed and individual assays were utilized (Table S1). Bead-based immunoassays targeting ErbB2, EGFR, Cytokeratin 19(Cyfra 21-1), mesothelin (MSLN), AFP, CA 72-4, MICA, SCC, HSP 70, EPCAM, PSA, transthyretin (TTR), angiostatin (AS), thrombospondin (TSP), endostatin (ES), TgII, HE4, CA 15-3, CA 125, CEA, and Kallikrein 10 (Klk10) were developed by the UPCI Luminex Core Facility according to strict quality control standards[32]. Assays for MMP 1, 2, 3, and 7 and TIMP 1–4 were obtained from R&D Systems (Minneapolis, MN). Assays for PDGF-BB, RANTES, SCGF-B, and NGF were obtained from Bio-Rad (Hercules, CA). Assays for HSP27, IL-1α, IL-1β, IL-3, IL-5, IL-7, IL-8, IL-12p40, IL-15, IL-17, TNF-α, IFNα, GM-CSF, MCP-1, MCP-3, MIP-1α, MIP-1β, IP-10, Eotaxin (CCL11), RANTES, DR5, EGF, FGFb, G-CSF, HGF, VEGF, and GROα were obtained from Life Technologies/Biosource (Grand Island, NY). All other assays were obtained from Merck/Millipore (Durmstadt, Germany). All commercial immunoassays were performed according to manufacturer's protocols while UPCI Luminex Core Facility assays were performed as previously described [32], [33].

**Table 2:** Complete List of Evaluated Biomarkers.

**Tumor Markers**	AFP, CA 15-3, CA 19-9, CA125, CA72-4, CEA, EPCAM, HE4, Kallikrein-10 (Klk10), Mesothelin (MSLN), PSA, squamous cell carcinoma antigen (SCC), tissue transglutaminase (Tgll)
**Growth/Angiogenesis Factors**	Angiostatin (AS), EGF, EGFR, Endostatin (ES), ErbB2, FGFb, HGF, IGFBP 1-7, NGF, PDGF-BB, SCGF-B, sVEGF-1, sVEGFR2-3, TGFα, Thrombospondin (TSP)
**Hormones**	ACTH, Cortisol, FSH, GH, Insulin, LH, Prolactin, PTH, TSH
**Apoptosis Factors**	Cyfra 21-1, Fas, FasL
**Cytokines/Chemokines/Receptors**	6CKine/CCL21, CTACK/CCL27, DR5, ENA-78/CXCL5, EOTAXIN-1 (CCL11), EOTAXIN-2/CCl24/MPIF-2, EOTAXIN-3/CCL26, FLT3, Fractalkine, GCP-2/CXCL6/LIX, G-CSF, GM-CSF, GROα, HCC-1/CCL14a, I-309/CCL1, IFNα, IL-1α, IL-1β, IL-3,5,7,8,11, IL-12p70, IL-12p40, IL-15-17,20, IL-28α, IL-29, IL-33/NF-HEV(mat), IP-10, I-TAC/CXCL11, LIF, Lymphotactin, MCP-1-4, M-CSF, MDC, MIF, MIP-1α, MIP-1β, MIP-1δ/MIP-5/CCL15, MIP-3α/CCL20, MIP-3β/CCL19, MIP-4, MPO, NAP-2/CXCL7, RANTES, sCD40L, SCF, SDF-1a+b/CXCL12, sIL-1Rl,II, sIL-4R, sIL-6R, sRAGE, sTNF –Rl, sTNF-Rll, TARC/CCL17, TNFα, TNFβ, TPO, TRAIL/TNFSF10, TSLP
**Adhesion Molecules**	E-Selectin, Fibronectin (FN), sICAM, sVCAM
**MMPs/TIMPs**	MMP-1-3, 7-9, 12,13, TIMP-1-4
**Bone Factors**	Osteoprotegerin (OPG), Osteocalcin (OC), Osteopontin (OPN), RANKL
**Apolipoproteins**	Apo A1, Apo Clll, Apo E
**Acute Phase Proteins**	α1-Antitrypsin (A1A), α2-Macroglobulin, Complement C3 (C3), Complement C4, Complement Factor H (CFH), Fibrinogen (FBN), Haptoglobin (HPN), human serum albumin (HSA), transthyretin (TTR), serum amyloid A (SAA), serum amyloid P (SAP)
**Adipokines**	Adiponectin, aPAI-1, Leptin, Resistin, tPAI-1
**Other**	HSP 70, Involucrin, pan-Keratin (1,10,11), Keratin-6, LPS, MICA, PEDF

### Collection and statistical analysis of biomarker data

Each serum sample was analyzed for each multiplex immunoassay using the Bio-Plex suspension array system and the Bio-Plex Manager software (version 4.1) (Bio-Rad Laboratories, Hercules, CA). For each analyte, 100 beads were analyzed and the median fluorescence intensity was determined. Analysis of serum sample biomarker data was performed using five-parameter logistic curve fitting to standard analyte values. A number of samples fell below lower limit of extrapolation of the Bio-Plex Manager software for certain analytes and were therefore reported as out of range low (OOR <). The percentages of cases and controls classified in this manner for each analyte are listed in Table S2. Observed concentration values were assigned to each of the OOR < samples by manual extrapolation using the curve fitting parameters determined by the Bio-Plex software. Hence, no samples were excluded from the analysis on the basis of undetectability. The entire biomarker dataset has been deposited with the Center for Analysis and Management of Multicenter AIDS Cohort Study (CAMACS).

Biomarker distributions among the A-NHL and control groups were analyzed using both parametric and non-parametric statistical tests. In each analysis the minimum level of significance was p<0.05. For the parametric analysis, exact conditional logistic regression was performed on the biomarker data obtained for the A-NHL and control groups (n = 46 matched case-control pairs). The results were further analyzed using the Bonferroni Step-Down (Holm) correction for multiple comparisons. For the non-parametric analysis, the Mann-Whitney U test was utilized on data from 47 cases and 48 controls without regards to matching. Here, the False Discovery Rate (FDR) was controlled at 5% according to the method described by Benjamini and Hochberg[34]. Biomarker variations among several subtypes of NHL were evaluated using a one-way ANOVA with Tukey's multiple comparison test. Correlations between biomarker levels and HIV viral load were evaluated using the Spearman test for correlation.

### Pathway Analysis

The Ingenuity Pathway Analysis (IPA) software package (Ingenuity Systems, Inc., Redwood City, CA) was used to examine pathways and interactions associated with the most promising biomarkers identified in our analysis described above. Additional serum biomarkers demonstrating associations with A-NHL in previous reports, which were not measured in the current investigation, were also included in this analysis. The list of additional biomarkers included IL-6 [21], IL-10 [21], [23], [30], sCD30 [21], [24], sCD23 [21], sCD44 [26], sCD27 [21], CRP [21], and CXCL13 [27], [28]. Some of these reports utilized a number of overlapping MACS NHL cases as described above. The IPA analysis identifies interactions based solely on the identity of the biomarkers inputted into the software and did not utilize biomarker concentrations.

## Results

### Biomarker alterations among A-NHL cases and controls

The non-parametric analysis of biomarker distributions among all cases and controls revealed that 19 out of the 161 biomarkers tested were significantly altered in the comparison of A-NHL cases and controls (Table 3). Among these 19 significant biomarkers, two (MMP-9, OC) were observed to be decreased in the A-NHL group in comparison to the controls, while the remaining 17 were increased. The parametric statistical analysis on 46 case-control pairs yielded a larger list of 37 significant biomarkers (Table 4) which included all but two biomarkers (FBN, A1A) identified in the non-parametric analysis. Among the 37 significant biomarkers in this analysis, four (OC, ApoA1, MMP-9, IGFBP-6) were observed to be decreased in the A-NHL subjects relative to the control groups, while the remaining 33 biomarkers were all increased. All trends in biomarker alterations between the cases and controls were consistent among the non-parametric and parametric analyses despite the differential use of median and mean values, respectively, and slightly different numbers of subjects. The application of the Bonferroni Step-down (Holm) correction to our parametric analyses yielded a restricted list of three biomarkers (IL-11, CXCL11, IL-29) which were observed to be highly significant (Table 4). A comparison of the non-parametric and parametric analyses prior to Bonferroni correction (overlap between tables 3 and 4) yielded a consensus list of 17 serum biomarkers significantly altered in the comparison of the A-NHL and control groups. The consensus list included cytokines/chemokines (CCL19, CXCL11, MCP-2, MIP-1δ, IFNα, IL-11, IL-29, IP-10, M-CSF, sIL-1R1), acute phase proteins (C3, HPN, SAA, SAP), tissue remodeling factors (MMP-9, TIMP-1), and the bone metabolic factor OC.

**Table 3:** Serum biomarker levels in AIDS-NHL subjects and controls evaluated by Non-parametric Statistics.

	A-NHL (n = 47)		Control (n = 48)		A-NHL vs. Control	
Biomarker	Median	IQR	Median	IQR	MWU p value (trend)	
**CCL19/MIP-3β**	238	133	197	112	6.89E-03	(I)
**C3** †	111	53.7	80.9	33.0	1.52E-03	(I)
**CXCL11/I-TAC**	266	393	132	128	9.31E-05	(I)
FBN†	161	151	84.6	95.1	8.20E-04	(I)
**HPN** †	4054	3284	3041	2546	8.23E-03	(I)
**IFN-α**	34.1	43.6	20.5	13.9	1.89E-03	(I)
**IL-11**	278	163	146	145	9.97E-06	(I)
**IL-29/IFNλ1**	284	193	200	100	6.97E-04	(I)
**IP-10**	98.2	96.0	60.2	53.4	1.48E-03	(I)
**MCP-2**	71.3	56.1	53.5	35.8	8.32E-03	(I)
**M-CSF**	317	554	86.8	250	2.45E-03	(I)
**MIP-1δ**	3720	2267	2816	1941	8.05E-03	(I)
**MMP-9** †	81.0	76.5	128	133	5.88E-03	(D)
**OC**	3811	2644	4536	2808	7.79E-03	(D)
**SAA** †	591	3361	295	462	9.69E-03	(I)
**SAP** †	4934	1962	3647	1207	2.69E-04	(I)
**sIL-1R1**	26.8	15.7	21.1	8.10	7.37E-03	(I)
**TIMP-1** †	160	50.6	137	33.8	4.57E-03	(I)
A1A†	1769	2124	1062	810	3.28E-03	(I)

IQR – interquartile range; MWU - Mann Whitney U test with 5% False Discovery Rate; Biomarkers in bold represent consensus markers also selected by parametric statistics;

† values expressed in ng/ml, all others expressed in pg/ml; Trends: I – increased in A-NHL group, D – decreased in A-NHL group.

**Table 4:** Conditional Logistic Regression Analyses with Bonferroni Step-down Adjustment of Serum Biomarker Levels of AIDS-NHL and control subjects.

Biomarker	A-NHL (n = 46)		Controls (n = 46)		Exact p-value	Bonferroni Adjusted p-value
	Mean	SD	Mean	SD		
ApoA1	1267	252	1374	213	0.0111	NS
CA 19-9	4.97	26.9	0.490	0.197	0.0013	NS
**CCL19/MIP-3β**	648	2777	196	77.5	0.0025	NS
CCL20/MIP-3α	47.2	188	11.5	7.51	0.0056	NS
**C3**	111	47.3	81.9	30.3	0.0003	NS
CFH	559	169	473	94.8	0.0016	NS
**CXCL11/I-TAC**	436	535	175	151	0.0001	0.017
CXCL6/GCP-2	342	1362	114	51.7	0.0138	NS
CCL11	212	145	190	70.7	0.0061	NS
G-CSF	176	325	73.8	71.9	0.0023	NS
**HPN** †	5110	4037	3148	2284	0.0093	NS
I-309	11.3	29.9	3.39	3.52	0.0126	NS
**IFNα**	13365	87796	65.5	189	0.0407	NS
IGFBP-3†	1524	248	1420	328	0.0085	NS
IGFBP-5†	120	72.6	90.8	58.3	0.0124	NS
IGFBP-6†	149	42.0	169	64.0	0.0274	NS
**IL-11**	5663	36435	176	97.9	<0.0001	<0.017
IL-20	115	253	56.8	34.3	0.0177	NS
IL-28A	555	3128	16.0	20.4	0.0249	NS
**IL-29**	11685	76491	232	127	0.0001	0.017
IL-7	49.7	146	18.4	7.37	0.0066	NS
**IP-10**	130	93.0	86.8	91.4	0.0173	NS
**MCP-2**	88.0	66.9	61.5	36.1	0.025	NS
**M-CSF**	15887	105262	277	405	0.0065	NS
MIF	964	2916	246	181	0.0402	NS
**MIP-1δ**	5617	7942	3295	2265	0.0056	NS
MIP-4†	22.2	19.4	16.0	7.88	0.0205	NS
MMP-3†	57.5	163	18.1	7.30	0.0453	NS
**MMP-9** †	108	94.1	154	103	0.0171	NS
**OC**	3996	2421	5375	3116	0.006	NS
PEDF†	1777	424	1604	339	0.0379	NS
**SAA** †	18574	67358	582	1020	0.0008	NS
**SAP** †	4759	1623	3867	1307	0.004	NS
SCGF-B†	60.3	49.4	45.9	15.7	0.0464	NS
**sIL-1RI**	45.2	106	24.3	8.43	0.0041	NS
sTNF-RII†	10.5	4.56	8.43	3.56	0.0172	NS
**TIMP-1** †	199	174	148	40.4	0.0096	NS

SD – standard deviation, NS - non-significant;

† concentration expressed as ng/ml, all others expressed in pg/ml; Biomarkers in bold represent consensus markers also selected by non-parametric statistics.

### Correlation with HIV viral load

Biomarker measurements from the A-NHL and control groups were combined in order to identify correlations between specific biomarkers and HIV viral load. Among the complete panel of 161 biomarkers, a total of 17 biomarkers exhibited significant correlations with HIV viral load (data not shown). Most notably, 11 of the significantly correlated biomarkers were among the consensus biomarkers described above, and are presented in Table 5. With the notable exception of MMP-9, each of the significant correlations among the consensus biomarkers was consistent with HIV progression, i.e., higher biomarker concentrations were correlated with higher HIV viral load.

**Table 5:** Spearman Correlations of Consensus Biomarkers and HIV Load among all subjects.*

Biomarker	Spearman *r*	*p*
CCL19/Mip-3β	0.67	0.0001
CXCL11/I-TAC	0.52	0.0001
IL-11	0.50	0.0001
IP-10	0.46	0.0003
MCP-2	0.42	0.001
IFN-α	0.42	0.0011
M-CSF	0.41	0.0013
SAP	0.40	0.0019
MMP-9	−0.38	0.0029

^*^Analysis included all subjects with available HIV load measurement (n = 58).

### A-NHL subtype specific biomarker alterations

Each of the 17 consensus biomarkers were evaluated in case subsets including subjects diagnosed with B-cell diffuse, CNS, or Burkitt's Lymphoma (BL)/BL-like NHL using pair-wise one-way ANOVA. As this analysis included cases only, biomarker levels were normalized to CD4 counts prior to ANOVA to account for variations in disease severity. In a comparison of BL/BL-like and CNS NHL cases, OC and Timp-1 were significantly different with p<0.05. In the comparison of CNS and B-cell diffuse NHL cases, three significant alterations were observed including OC, SAP and Timp-1 (data not shown). In each comparison, significantly elevated levels of each biomarker were observed in CNS NHL relative to the other subtypes.

### Pathway Analysis

**Figure 1:**
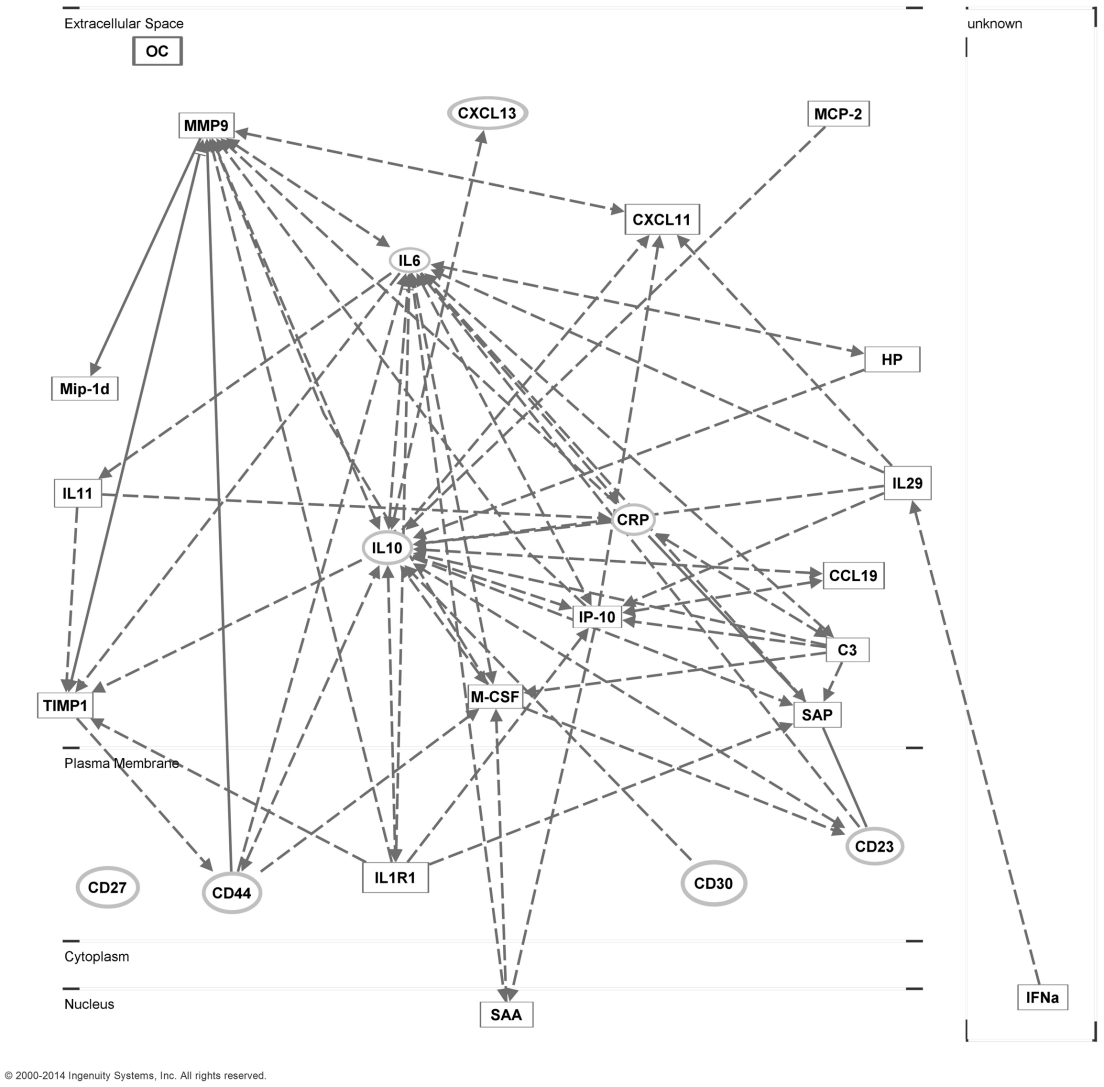
A-NHL biomarker pathway analysis. The Ingenuity Pathway Analysis (IPA) software package was utilized to identify pathways and specific interactions associated with the serum biomarkers identified in the current and previous reports. Biomarkers evaluated from the current study (rectangles) included CCL19, CXCL11, MCP-2, MIP-1δ, IFN-α, IL-11, IL-29, IP-10, M-CSF, sIL-1R1, C3, HPN, SAA, SAP, MMP-9, TIMP-1 and OC. Biomarkers evaluated based on previous findings (ovals) included IL-6, sCD30, IL-10, sCD23, sCD44, sCD27, CRP, BCA-1/CXCL13 and IgE. Solid lines indicate direct interactions, dashed lines indicate indirect interactions.

The IPA software package was first used to analyze the consensus list of 17 serum biomarkers identified in this study. The top biological functions associated with the consensus list included Cellular Movement (14/17 molecules, p = 1.6×10^−14^–1.9×10^−3^), Cell-to-Cell Signaling and Interaction (13/17 molecules, p = 1.4×10^−10^–1.9×10^−3^), Hematological System Development and Function (14/17 molecules, p = 1.6×10^−14^–1.9×10^−3^), and Immune Cell Trafficking (14/17 molecules, p = 1.6×10^-14^–1.9×10^−3^). Interactions between molecules were then examined for each biomarker included in the consensus list and several biomarkers examined in association with A-NHL in previous studies including IL-6, sCD30, IL-10, sCD23, sCD44, sCD27, CRP, CXCL13, and IgE. An extensive array of interactions within and among the two lists of biomarkers was identified (Figure 1). With the exceptions of MIP-1δ, IFNα, and OC, each of the newly identified biomarkers was found to interact with at least one previously identified biomarker. MMP-9 demonstrated the most extensive network of interactions including six interactions with other newly identified biomarkers and four interactions with previously identified biomarkers. Other newly identified biomarkers demonstrating more than five interactions included IP-10 (7 new/2 previous), M-CSF (3 new/4 previous), sIL-1R1 (4 new/2 previous), TIMP-1 (3 new/3 previous), C3 (3 new/3 previous), and SAP (2 new/4 previous). Many of the previously identified biomarkers also displayed numerous interactions within and among the groups.

## Discussion

A diverse analysis of serum proteins in samples collected just before or after the time of A-NHL diagnosis identified a consensus list of potential A-NHL biomarkers, assembled through rigorous statistical analysis, which included various inflammatory mediators, acute phase proteins and tissue remodeling agents. Although our panel of candidate biomarkers was considerably large and composed primarily of inflammatory and immune-modulating factors, a relatively small subset was observed to be altered, suggestive of some level of specificity for A-NHL with respect to HIV+ status or other AIDS defining illness. Increased levels of a number of circulating proteins have previously been reported up to 3–5 years prior to the diagnosis of A-NHL including IL-6, IL-10, sCD23, sCD27, sCD30, sCD44, IgE, CRP, and BCA-1/CXCL13[21]–[24], [26]–[28], [30], [35], [36]. While the current study did not include a direct examination of these biomarkers, an analysis of biomarker interactions revealed a number of mechanistic links between and among those biomarkers identified both previously and currently (Figure 1). The most recent longitudinal study of A-NHL risk in HIV+ persons identified IP-10 and TNFα, in addition to several others, as potential biomarkers [22]. The current cross-sectional analysis evaluated both of these biomarkers but found only IP-10 to be significantly altered.

The conservative use of the Bonferroni correction as part of our parametric statistical analysis suggested that serum levels of IL-11, CXCL11 and IL-29 demonstrated the strongest associations with A-NHL in our study. These findings further agree with the hypothesized immune activating origins of this disease. IL-11, an important mediator of hematopoiesis, has been investigated as an experimental treatment for hematological malignancies [37]. CXCL11 is a chemokine known to mediate the migration of activated T-cells, and has been described as an immunomodulatory target of miRNA in primary lymphoma and has also demonstrated potent antitumor activity in an animal model [38], [39]. The type III interferon IL-29 is the focus of considerable interest in the setting of hepatitis C viral infection and was recently shown to impede HIV-1 infection in T-cells [40], [41]. A role in the development of A-NHL for IL-29 has not yet been described.

A number of additional proteins with varying biological functions but unifying roles in inflammation and immune activation were also associated with A-NHL in univariate analyses in the current study, but were not statistically significant after Bonferroni correction. In such a broad survey, this is not unexpected, but the data does confirm previous reports of some of these proteins being associated with inflammatory processes around the time of A-NHL or NHL diagnosis. Several of the chemoattractant proteins (CCL19/MIP-3β, MCP-2, CXCL9/MIG) have been reported to have an association with various forms of leukemia and lymphoma [42], [43], ^41^. Of particular interest for A-NHL is IP-10, a leukocyte chemoattractant implicated in the development and progression of B-cell lymphoma. In two recent studies, elevated levels of IP-10 in the sera of HIV-positive individuals were associated with the development of B-cell lymphoma within 0–5 years ^22^, [44]. Elevated pretreatment serum levels of IP-10 were also associated with increased likelihood of disease relapse and diminished survival in HIV-uninfected patients with diffuse large B-cell lymphoma [45]. The gelatinase MMP-9 and its inhibitor TIMP-1 mediate breakdown of the extracellular matrix during inflammatory and other responses, and one or both have been linked to lymphoma at the time of diagnosis [46] [47], [48]. The acute phase protein, SAA has been shown to be overexpressed in B-cell NHL associated with SIV infection in monkeys [49], and complement components (C3 and CR2) may be involved in lymphomagenesis and/or complement activation [50], [51]. IFNα is currently under investigation as a potential therapeutic agent in lymphoma, including A-NHL, stemming from its ability to mediate apoptosis in malignant cells[52]–[54].

Biological roles related to the development of lymphoma have not been conclusively described for MIP-1δ, M-CSF, sIL-1R1, HPN, SAP, and OC and, to the best of our knowledge, we are the first to report on these associations. Each of these proteins does possess functions relating them to inflammation and were linked mechanistically to other potential biomarkers in our pathway analysis. Further investigation into the precise activity of each of these proteins in lymphoma would be of great interest.

Many of the biomarkers found to be significantly altered in A-NHL subjects appear to be positively associated with HIV disease progression, as indicated by their observed correlation with HIV viral load (Table 5). Each of the biomarkers found to be positively correlated with viral load were also observed to be elevated in the sera of A-NHL cases in comparison to the controls. As CD4 count was included in our matching criteria and was not significantly different between the two experimental groups, we propose that varying degrees of control of HIV (or lack thereof) within each experimental group, as opposed to among the groups, led to our observations. These observations are in line with the hypothesis that increases in HIV viral burden result in elevated levels of immune stimulation and inflammation which then promotes NHL pathogenesis. Based on these findings, we may also hypothesize that HIV-driven alterations in immune response are augmented in A-NHL, a phenomenon that warrants additional investigation. In contrast to other biomarkers, higher MMP-9 levels correlated with lower viral load and control (non-malignancy) status. A direct relationship between increased MMP-9 activity and immune competence and/or protection from lymphoma has not been described, leading us to speculate that the relationship between MMP-9 and A-NHL may be a protective one which warrants further exploration.

When A-NHL subjects of differing disease subtypes were evaluated separately, relatively few significant alterations were found. Several biomarkers including OC, SAP, and Timp-1 were found to be significantly elevated in CNS NHL in comparison to other subtypes. To the best of our knowledge, we are the first to report on such associations. For the vast majority of the biomarkers examined, we were not able to detect any significant differences between NHL subtypes, although this may be due to the relatively small sample size of each subset and the amount of variability within both case and control groups. Our results are similar to the recent findings of Breen et al. in which no significant differences were observed when comparing biomarker levels of diffuse large B cell cases to BL cases; however, in that study all significantly increased biomarker levels in cases compared to controls were associated with the systemic form of NHL but not CNS NHL [21]. While utilizing many of the same MACS A-NHL cases, the current investigation in general examined different time points (closer to or after NHL diagnosis), and each study evaluated a distinct set of biomarkers.

The results of the pathway analysis indicate the existence of a complex network of interactions among the collective list of biomarkers identified in the current and previous investigations. An examination of this network suggests central roles for two previously identified biomarkers, IL-6 and IL-10 (Figure 1). Several newly identified biomarkers including MMP-9, IP-10, and M-CSF also appear to play central roles. Each of the three biomarkers demonstrating the strongest associations with A-NHL in the current study (IL-11, CXCL11, and IL-29) were found to interact with biomarkers from both the current and previous reports. In the course of further development of potential A-NHL biomarkers, many candidate proteins are likely to be eliminated based on technical and/or biological limitations. A more complete understanding of the network of interactions displayed in this analysis should enable us to more effectively identify potential alternatives to those biomarkers which work in close proximity within disease processes.

In the current study, an analysis of a broad array of circulating proteins yielded a relatively small but robust collection of inflammatory mediators associated with the development of A-NHL in comparison to controls. Many of the identified biomarkers were positively correlated to HIV viral load and indicate heightened immune responses in A-NHL with respect to HIV infection and/or AIDS in the absence of lymphoma. A minority of the subjects included in the current study were former or current recipients of HAART. Although the distribution of HAART recipients was similar among cases and controls, we cannot rule out some effect of treatment on our biomarker findings. Our findings further support the hypothesis that A-NHL develops in response to persistent immune activation and inflammation and adds new points of focus for further investigation. Ongoing analysis into the precise role of each of the biomarkers identified in our report should enhance our ability to diagnose, monitor and treat this disease.

## Supporting Information

Table S1 **Description of Biomarker assays utilized in the current study.**
(DOCX)

Table S2 **Percentage of samples classified as out of range low according to the Bioplex Manager software.**
(DOCX)

## References

[1] Grulich AE, Li Y, McDonald AM, Correll PK, Law MG, et al (2001) Decreasing rates of Kaposi's sarcoma and non-Hodgkin's lymphoma in the era of potent combination anti-retroviral therapy. AIDS 15: 629–633. 11317001 10.1097/00002030-200103300-00013

[2] Bonnet F, Balestre E, Thiebaut R, Morlat P, Pellegrin JL, et al (2006) Factors associated with the occurrence of AIDS-related non-Hodgkin lymphoma in the era of highly active antiretroviral therapy: Aquitaine Cohort, France. Clin Infect Dis 42: 411–417. 16392091 10.1086/499054

[3] Bonnet F, Lewden C, May T, Heripret L, Jougla E, et al (2004) Malignancy-related causes of death in human immunodeficiency virus-infected patients in the era of highly active antiretroviral therapy. Cancer 101: 317–324. 15241829 10.1002/cncr.20354

[4] Matthews GV, Bower M, Mandalia S, Powles T, Nelson MR, et al (2000) Changes in acquired immunodeficiency syndrome-related lymphoma since the introduction of highly active antiretroviral therapy. Blood 96: 2730–2734. 11023505

[5] Beral V, Peterman T, Berkelman R, Jaffe H (1991) AIDS-associated non-Hodgkin lymphoma. Lancet 337: 805–809. 1672911 10.1016/0140-6736(91)92513-2

[6] Rabkin CS (2001) AIDS and cancer in the era of highly active antiretroviral therapy (HAART). European Journal of Cancer 37: 1316–1319. 11423263 10.1016/s0959-8049(01)00104-6

[7] Seaberg EC, Wiley D, Martinez-Maza O, Chmiel JS, Kingsley L, et al (2010) Cancer incidence in the multicenter AIDS Cohort Study before and during the HAART era: 1984 to 2007. Cancer 116: 5507–5516. 20672354 10.1002/cncr.25530 PMC2991510

[8] Beral V, Jaffe HW, Weiss RA (1991) Cancer, HIV and AIDS. Cancer Surveys: 1–5. 1821317

[9] Bower M, Fife K (2001) Current issues in the biology of AIDS-related lymphoma. HIV Medicine 2: 141–145. 11737393 10.1046/j.1468-1293.2001.00061.x

[10] Cote TR, Manns A, Hardy CR, Yellin FJ, Hartge P (1996) Epidemiology of brain lymphoma among people with or without acquired immunodeficiency syndrome. AIDS/Cancer Study Group. J Natl Cancer Inst 88: 675–679. 8627644 10.1093/jnci/88.10.675

[11] Knowles DM (2003) Etiology and pathogenesis of AIDS-related non-Hodgkin's lymphoma. Hematology/Oncology Clinics of North America 17: 785–820. 12852656 10.1016/s0889-8588(03)00050-9

[12] Polesel J, Clifford GM, Rickenbach M, Dal Maso L, Battegay M, et al (2008) Non-Hodgkin lymphoma incidence in the Swiss HIV Cohort Study before and after highly active antiretroviral therapy. AIDS 22: 301–306. 18097233 10.1097/QAD.0b013e3282f2705d

[13] Epeldegui M, Widney DP, Martinez-Maza O (2006) Pathogenesis of AIDS lymphoma: role of oncogenic viruses and B cell activation-associated molecular lesions. Curr Opin Oncol 18: 444–448. 16894291 10.1097/01.cco.0000239882.23839.e5

[14] Carbone A (2002) AIDS-related non-Hodgkin's lymphomas: From pathology and molecular pathogenesis to treatment. Human Pathology 33: 392–404. 12055673 10.1053/hupa.2002.124723

[15] Gaidano G, Carbone A, Dalla-Favera R (1998) Pathogenesis of AIDS-related lymphomas: Molecular and histogenetic heterogeneity. American Journal of Pathology 152: 623–630. 9502401 PMC1858382

[16] Cunto-Amesty G, Przybylski G, Honczarenko M, Monroe JG, Silberstein LE (2000) Blood 95: 1393–1399. 10666216

[17] Grulich AE (2000) Update: Cancer risk in persons with HIV/AIDS in the era of combination antiretroviral therapy. AIDS Reader 10: 341–346. 10881365

[18] Grulich AE, Wan X, Law MG, Milliken ST, Lewis CR, et al (2000) B-cell stimulation and prolonged immune deficiency are risk factors for non-Hodgkin's lymphoma in people with AIDS. AIDS 14: 133–140. 10708283 10.1097/00002030-200001280-00008

[19] Kirk O, Pedersen C, Cozzi-Lepri A, Antunes F, Miller V, et al (2001) Non-Hodgkin lymphoma in HIV-infected patients in the era of highly active antiretroviral therapy. Blood 98: 3406–3412. 11719381 10.1182/blood.v98.12.3406

[20] Killebrew D, Shiramizu B (2004) Pathogenesis of HIV-associated non-Hodgkin lymphoma. Curr HIV Res 2: 215–221. 15279585 10.2174/1570162043351237

[21] Breen EC, Hussain SK, Magpantay L, Jacobson LP, Detels R, et al (2011) B-cell stimulatory cytokines and markers of immune activation are elevated several years prior to the diagnosis of systemic AIDS-associated non-Hodgkin B-cell lymphoma. Cancer Epidemiol Biomarkers Prev 20: 1303–1314. 21527584 10.1158/1055-9965.EPI-11-0037 PMC3132317

[22] Vendrame E, Hussain SK, Breen EC, Magpantay LI, Widney DP, et al (2014) Serum Levels of Cytokines and Biomarkers for Inflammation and Immune Activation, and HIV-Associated Non-Hodgkin B-Cell Lymphoma Risk. Cancer Epidemiol Biomarkers Prev 23: 343–349. 24220912 10.1158/1055-9965.EPI-13-0714 PMC3948172

[23] Breen EC, Boscardin WJ, Detels R, Jacobson LP, Smith MW, et al (2003) Non-Hodgkin's B cell lymphoma in persons with acquired immunodeficiency syndrome is associated with increased serum levels of IL10, or the IL10 promoter -592 C/C genotype. Clin Immunol 109: 119–129. 14597210 10.1016/s1521-6616(03)00214-6

[24] Breen EC, Fatahi S, Epeldegui M, Boscardin WJ, Detels R, et al (2006) Elevated serum soluble CD30 precedes the development of AIDS-associated non-Hodgkin's B cell lymphoma. Tumor Biology 27: 187–194. 16651853 10.1159/000093022

[25] Schroeder JR, Saah AJ, Hoover DR, Margolick JB, Ambinder RF, et al (1999) Serum soluble CD23 level correlates with subsequent development of AIDS-related non-Hodgkin's lymphoma. Cancer Epidemiol Biomarkers Prev 8: 979–984. 10566552

[26] Breen EC, Epeldegui M, Boscardin WJ, Widney DP, Detels R, et al (2005) Elevated levels of soluble CD44 precede the development of AIDS-associated non-Hodgkin's B-cell lymphoma [4]. AIDS 19: 1711–1712. 16184051 10.1097/01.aids.0000184924.04983.7c

[27] Hussain S, Zhu W, Chang SC, Crabb Breen E, Vendrame E, et al (2013) Serum levels of the chemokine CXCL13, genetic variation in CXCL13 and its receptor CXCR5, and HIV-associated non-Hodgkin B cell lymphoma risk. Cancer Epidemiol Biomarkers Prev 22: 295–307. 23250934 10.1158/1055-9965.EPI-12-1122 PMC3703445

[28] Widney DP, Gui D, Popoviciu LM, Said JW, Breen EC, et al (2010) Expression and Function of the Chemokine, CXCL13, and Its Receptor, CXCR5, in Aids-Associated Non-Hodgkin's Lymphoma. AIDS Res Treat 2010: 164586. 21490903 10.1155/2010/164586 PMC3065842

[29] Kaslow RA, Ostrow DG, Detels R, Phair JP, Polk BF, et al (1987) The Multicenter AIDS Cohort Study: rationale, organization, and selected characteristics of the participants. Am J Epidemiol 126: 310–318. 3300281 10.1093/aje/126.2.310

[30] Wong HL, Breen EC, Pfeiffer RM, Aissani B, Martinson JJ, et al (2010) Cytokine signaling pathway polymorphisms and AIDS-related non-Hodgkin lymphoma risk in the multicenter AIDS cohort study. AIDS 24: 1025–1033. 20299965 10.1097/QAD.0b013e328332d5b1 PMC3950937

[31] (2008) Panel on Antiretroviral Guidelines for Adults and Adolescents. Guidelines for the use of antiretroviral agents in HIV-1-infected adults and adolescents. Department of Health and Human Services.

[32] UPCI Luminex Core Facility. http://www.upci.upmc.edu/cbf/luminex.cfm

[33] Yurkovetsky Z, Skates S, Lomakin A, Nolen B, Pulsipher T, et al (2010) Development of a multimarker assay for early detection of ovarian cancer. J Clin Oncol 28: 2159–2166. 20368574 10.1200/JCO.2008.19.2484 PMC2860434

[34] Benjamini Y, Hochberg Y (1995) Controlling the false discovery rate: a practical and powerful approach to multiple testing. Journal of the Royal Statistical Society, Series B (Methodological) 57: 289–300.

[35] Pagès F, Galon J, Karaschuk G, Dudziak D, Camus M, et al (2005) Epstein-Barr virus nuclear antigen 2 induces interleukin-18 receptor expression in B cells. Blood 105: 1632–1639. 15498855 10.1182/blood-2004-08-3196

[36] Purdue MP, Lan Q, Martinez-Maza O, Oken MM, Hocking W, et al (2009) A prospective study of serum soluble CD30 concentration and risk of non-Hodgkin lymphoma. Blood 114: 2730–2732. 19638620 10.1182/blood-2009-04-217521 PMC2756127

[37] Ellis M, Hedstrom U, Frampton C, Alizadeh H, Kristensen J, et al (2006) Modulation of the systemic inflammatory response by recombinant human interleukin-11: a prospective randomized placebo controlled clinical study in patients with hematological malignancy. Clin Immunol 120: 129–137. 16644288 10.1016/j.clim.2006.03.003

[38] Hensbergen PJ, Wijnands PG, Schreurs MW, Scheper RJ, Willemze R, et al (2005) The CXCR3 targeting chemokine CXCL11 has potent antitumor activity in vivo involving attraction of CD8+ T lymphocytes but not inhibition of angiogenesis. J Immunother 28: 343–351. 16000952 10.1097/01.cji.0000165355.26795.27

[39] Xia T, O'Hara A, Araujo I, Barreto J, Carvalho E, et al (2008) EBV microRNAs in primary lymphomas and targeting of CXCL-11 by ebv-mir-BHRF1-3. Cancer Res 68: 1436–1442. 18316607 10.1158/0008-5472.CAN-07-5126 PMC2855641

[40] Dolganiuc A, Kodys K, Marshall C, Saha B, Zhang S, et al (2012) Type III Interferons, IL-28 and IL-29, Are Increased in Chronic HCV Infection and Induce Myeloid Dendritic Cell-Mediated FoxP3+ Regulatory T Cells. PLoS One 7: e44915. 23071503 10.1371/journal.pone.0044915 PMC3468613

[41] Tian RR, Guo HX, Wei JF, Yang CK, He SH, et al (2012) IFN-lambda inhibits HIV-1 integration and post-transcriptional events in vitro, but there is only limited in vivo repression of viral production. Antiviral Res 95: 57–65. 22584351 10.1016/j.antiviral.2012.04.011

[42] Corcione A, Arduino N, Ferretti E, Raffaghello L, Roncella S, et al (2004) CCL19 and CXCL12 trigger in vitro chemotaxis of human mantle cell lymphoma B cells. Clin Cancer Res 10: 964–971. 14871974 10.1158/1078-0432.ccr-1182-3

[43] Wang X, Yuling H, Yanping J, Xinti T, Yaofang Y, et al (2007) CCL19 and CXCL13 synergistically regulate interaction between B cell acute lymphocytic leukemia CD23+CD5+ B Cells and CD8+ T cells. J Immunol 179: 2880–2888. 17709502 10.4049/jimmunol.179.5.2880

[44] Ouedraogo DE, Makinson A, Kuster N, Nagot N, Rubbo PA, et al (2013) Increased T-Cell Activation and Th1 Cytokine Concentrations Prior to the Diagnosis of B-Cell Lymphoma in HIV Infected Patients. J Clin Immunol 33: 22–29. 22914896 10.1007/s10875-012-9766-0

[45] Ansell SM, Maurer MJ, Ziesmer SC, Slager SL, Habermann TM, et al (2012) Elevated pretreatment serum levels of interferon-inducible protein-10 (CXCL10) predict disease relapse and prognosis in diffuse large B-cell lymphoma patients. Am J Hematol 87: 865–869. 22674570 10.1002/ajh.23259 PMC3429646

[46] Bozkurt C, Ertem U, Oksal A, Sahin G, Yuksek N, et al (2008) Expression of matrix metalloproteinase-9 (MMP-9) and tissue inhibitor of matrix metalloproteinase (TIMP-1) in tissues with a diagnosis of childhood lymphoma. Pediatr Hematol Oncol 25: 621–629. 18850474 10.1080/08880010802313657

[47] Citak EC, Oguz A, Karadeniz C, Akyurek N (2008) Role of gelatinases (MMP-2 and MMP-9), TIMP-1, vascular endothelial growth factor (VEGF), and microvessel density on the clinicopathological behavior of childhood non-Hodgkin lymphoma. Pediatr Hematol Oncol 25: 55–66. 18231955 10.1080/08880010701826866

[48] Hottinger AF, Iwamoto FM, Karimi S, Riedel E, Dantis J, et al (2011) YKL-40 and MMP-9 as serum markers for patients with primary central nervous system lymphoma. Ann Neurol 70: 163–169. 21391238 10.1002/ana.22360 PMC7295085

[49] Tarantul VZ, Nikolaev AI, Martynenko A, Hannig H, Hunsmann G, et al (2000) Differential gene expression in B-cell non-Hodgkin's lymphoma of SIV-infected monkey. AIDS Res Hum Retroviruses 16: 173–179. 10659056 10.1089/088922200309511

[50] Bassig BA, Zheng T, Zhang Y, Berndt SI, Holford TR, et al (2012) Polymorphisms in complement system genes and risk of non-Hodgkin lymphoma. Environ Mol Mutagen 53: 145–151. 22170086 10.1002/em.21675 PMC3391498

[51] Marquart HV, Olesen EH, Johnson AA, Damgaard G, Leslie RG (1997) A comparative study of normal B cells and the EBV-positive Burkitt's lymphoma cell line, Raji, as activators of the complement system. Scand J Immunol 46: 246–253. 9315112 10.1046/j.1365-3083.1997.d01-122.x

[52] Harrington WJ Jr, Cabral L, Cai JP, Chan ASS, Wood C (1996) Azothymidine and interferon-alpha are active in AIDS-associated small non-cleaved cell lymphoma but not large-cell lymphoma. Lancet 348: 833. 10.1016/s0140-6736(05)65260-9 8814017

[53] Lee RK, Cai JP, Deyev V, Gill PS, Cabral L, et al (1999) Azidothymidine and interferon-alpha induce apoptosis in herpesvirus-associated lymphomas. Cancer Res 59: 5514–5520. 10554028

[54] Toomey NL, Deyev VV, Wood C, Boise LH, Scott D, et al (2001) Induction of a TRAIL-mediated suicide program by interferon alpha in primary effusion lymphoma. Oncogene 20: 7029–7040. 11704827 10.1038/sj.onc.1204895

