# Supplementary material for: Circulating Mediators of Inflammation and Immune Activation in AIDS-Related Non-Hodgkin Lymphoma
Source: PLoS One. 2014 Jun 12;9(6):e99144. doi: 10.1371/journal.pone.0099144 (PMC4055650; doi:10.1371/journal.pone.0099144)
Supplement: Table S1 — Description of Biomarker assays utilized in the current study. (DOCX) [file pone.0099144.s001.docx]

| **Supplementary Table S1. Biomarker assays utilized in the current study.** | | | |
| --- | --- | --- | --- |
| Panel # | Manufacturer | Dilution Factor | Analytes |
| 1 | UPCI | 1:5 | ErbB2, EGFR, Cytokeratin 19, CA 19-9, CA 125, CEA |
| 2 | UPCI | 1:5 | Mesothelin, AFP, Kallikrein 10 |
| 3 | UPCI | 1:5 | MICA, SCC, HSP 70, EPCAM, PSA |
| 4 | UPCI | 1:800 | TTR |
| 5 | UPCI | 1:300 | Angiostatin, Thrombospondin, Endostatin |
| 6 | UPCI | 1:3 | Tgll |
| 7 | UPCI | 1:5 | HE4, CA72-4, CA15-3 |
| 8 | R&D Systems | 1:5 | MMP-1, MMP-2, MMP-3, MMP-7, MMP-8, MMP-12, MMP-13 |
| 9 | R&D Systems | 1:50 | TIMP-1, TIMP-2, TIMP-3, TIMP-4 |
| 10 | Bio-Rad | 1:4 | PDGF-BB, RANTES, SCGF-B, NGF |
| 11 | Biosource | neat | IL-1α, IL-1β, IL-3, IL-5, IL-7, IL-8, IL-15, IL-17, TNFα, IFNα, GM-CSF, IL-12p40, IL-12p70, MCP-1, MCP-3, MIP-1α, MIP-1β, IP-10, EOTAXIN/CCL11, DR5, EGF, FGFb, G-CSF, HGF, GROα, |
| 12 | Millipore | 1:5 | ACTH, Insulin, Leptin, OPG, OC, OPN, PTH |
| 13 | Millipore | neat | 6CKine/CCL21, CTACK/CCL27, ENA-78/CXCL5, EOTAXIN-2/CCl24/MPIF-2, EOTAXIN-3/CCL26, I-309/CCL1, IL-16, IL-20, IL-28α, IL-33/NF-HEV, LIF, MCP-2, MCP-4, MIP-1δ(MIP-5/CCL15), SCF, SDF-1a+b/CXCL12, TARC/CCL17, TPO, TRAIL/TNFSF10, TSLP |
| 14 | Millipore | neat | GCP-2/CXCL6/LIX, IL-11, IL-29, I-TAC/CXCL11, Lymphotactin, M-CSF, MIP-3α/CCL20, MIP-3β/CCL19 |
| 15 | Millipore | 1:100 | HCC-1/CCL14a, NAP-2/CXCL7 |
| 16 | Millipore | 1:40000 | α2-Macroglobulin, Apo A1, Apo Clll, Apo E, Complement C3, Complement Factor H, Prealbumin |
| 17 | Millipore | 1:2000 | α1-Antitrypsin, Complement C4, MIP-4, PEDF |
| 18 | Millipore | neat | Cortisol, Fibronectin, Human Serum Albumin, Involucrin, LPS, Keratin-6, Keratin-1,10,11 |
| 19 | Millipore | 1:25 | IGFBP-1, IGFBP-2, IGFBP-3, IGFBP-4, IGFBP-5, IGFBP-6, IGFBP-7 |
| 20 | Millipore | 1:100 | sVCAM, sICAM, MPO, Adiponectin, tPAI-1, MMP-9, E-Selectin |
| 21 | Millipore | neat | FSH, LH, GH, TSH, Prolactin |
| 22 | Millipore | 1:400 | Resistin, aPAI-1 |
| 23 | Millipore | 1:2 | Fas, FasL, MIF |
| 24 | Millipore | neat | Fractalkine, sCD40L, TGFα, FLT3, MDC, TNFβ |
| 25 | Millipore | 1:5 | sIL-1Rl, sIL-1Rll, sIL-4R, sIL-6R, sRAGE, sTNF –Rl, sTNF-Rll, sVEGF-1, sVEGF-R2, sVEGF-R3 |
| 26 | Millipore | 1:2000 | Fibrinogen |
| 27 | Millipore | 1:40000 | Haptoglobin |
| 28 | Millipore | 1:2 | RANKL |
| 29 | Millipore | 1:2000 | SAA, SAP |
| UPCI – UPCI Luminex Core Facility  R&D Systems – Minneapolis, MN  Bio-Rad – Bio Rad Laboratories, Inc., Hercules, Ca  Biosource – Life Technologies, Grand Island, NY  Millipore – Merck/Millipore, Durmstradt, Germany | | | |
