# Supplementary material for: Circulating Mediators of Inflammation and Immune Activation in AIDS-Related Non-Hodgkin Lymphoma
Source: PLoS One. 2014 Jun 12;9(6):e99144. doi: 10.1371/journal.pone.0099144 (PMC4055650; doi:10.1371/journal.pone.0099144)
Supplement: Table S2 — Percentage of samples classified as out of range low according to the Bioplex Manager software. (DOCX) [file pone.0099144.s002.docx]

| **Supplementary Table S2. Percentage of samples classified as out of range low according to the Bioplex Manager software** | | | | | | | | |
| --- | --- | --- | --- | --- | --- | --- | --- | --- |
| **Analyte** | **Cases** | **Controls** | **Total** |  | **Analyte** | **Cases** | **Controls** | **Total** |
| 6Ckine | 0.00 | 0.00 | 0.00 |  | Kallikrein 10 | 0.00 | 0.00 | 0.00 |
| α1-Antitrypsin | 0.00 | 0.00 | 0.00 |  | Keratin-1,-10,-11 | 59.57 | 89.58 | 74.74 |
| α-2-Macroglobulin | 0.00 | 0.00 | 0.00 |  | Keratin-6 | 87.23 | 93.75 | 90.53 |
| ACTH | 57.45 | 60.42 | 58.95 |  | Leptin | 48.94 | 47.92 | 48.42 |
| Adiponectin | 0.00 | 0.00 | 0.00 |  | LH | 0.00 | 0.00 | 0.00 |
| AFP | 0.00 | 0.00 | 0.00 |  | LIF | 10.64 | 4.17 | 7.37 |
| Angiostatin | 0.00 | 0.00 | 0.00 |  | LPS | 42.55 | 41.67 | 42.11 |
| Haptoglobin | 0.00 | 0.00 | 0.00 |  | MCP-1 | 0.00 | 0.00 | 0.00 |
| Apo A1 | 0.00 | 0.00 | 0.00 |  | MCP-2 | 0.00 | 2.08 | 1.05 |
| Apo CIII | 0.00 | 0.00 | 0.00 |  | MCP-3 | 19.15 | 31.25 | 25.26 |
| Apo E | 0.00 | 0.00 | 0.00 |  | MCP-4 | 0.00 | 0.00 | 0.00 |
| b-NGF | 0.00 | 0.00 | 0.00 |  | M-CSF | 12.77 | 39.58 | 26.32 |
| CA 15-3 | 4.26 | 4.17 | 4.21 |  | MDC | 0.00 | 0.00 | 0.00 |
| CA 19-9 | 31.91 | 35.42 | 33.68 |  | Mesothelin | 0.00 | 0.00 | 0.00 |
| CA 72-4 | 4.26 | 0.00 | 2.11 |  | MICA | 70.21 | 87.50 | 78.95 |
| CA-125 | 0.00 | 0.00 | 0.00 |  | MIF | 0.00 | 2.08 | 1.05 |
| CCL14a/HCC-1 | 0.00 | 0.00 | 0.00 |  | MIP-1α | 0.00 | 0.00 | 0.00 |
| CCL19/MIP3β | 0.00 | 0.00 | 0.00 |  | MIP-1β | 0.00 | 0.00 | 0.00 |
| CCL20/MIP3α | 0.00 | 0.00 | 0.00 |  | MIP-1δ | 0.00 | 0.00 | 0.00 |
| CEA | 87.23 | 91.67 | 89.47 |  | MIP-4 | 0.00 | 0.00 | 0.00 |
| CFH | 0.00 | 0.00 | 0.00 |  | MMP-1 | 0.00 | 0.00 | 0.00 |
| Complement C3 | 0.00 | 0.00 | 0.00 |  | MMP-12 | 95.74 | 97.92 | 96.84 |
| Complement C4 | 0.00 | 0.00 | 0.00 |  | MMP-13 | 100.00 | 100.00 | 100.00 |
| Cortisol | 0.00 | 0.00 | 0.00 |  | MMP-2 | 0.00 | 0.00 | 0.00 |
| CTACK | 0.00 | 0.00 | 0.00 |  | MMP-3 | 0.00 | 0.00 | 0.00 |
| CXCL11/I-TAC | 0.00 | 0.00 | 0.00 |  | MMP-7 | 0.00 | 0.00 | 0.00 |
| CXCL6/ GCP2 | 0.00 | 0.00 | 0.00 |  | MMP-8 | 0.00 | 0.00 | 0.00 |
| CXCL7/NAP2 | 0.00 | 0.00 | 0.00 |  | MMP-9 | 0.00 | 0.00 | 0.00 |
| Cyfra 21-1 | 80.85 | 91.67 | 86.32 |  | MPO | 0.00 | 2.08 | 1.05 |
| DR5 | 36.17 | 35.42 | 35.79 |  | OC | 0.00 | 0.00 | 0.00 |
| EGF | 0.00 | 2.08 | 1.05 |  | OPG | 0.00 | 0.00 | 0.00 |
| EGFR | 0.00 | 0.00 | 0.00 |  | OPN | 21.28 | 16.67 | 18.95 |
| ENA-78 | 0.00 | 0.00 | 0.00 |  | aPAI-1 | 8.51 | 8.33 | 8.42 |
| Endostatin | 0.00 | 0.00 | 0.00 |  | PDGF-BB | 0.00 | 0.00 | 0.00 |
| EOTAXIN | 0.00 | 0.00 | 0.00 |  | PEDF | 0.00 | 0.00 | 0.00 |
| Eotaxin-2 | 0.00 | 0.00 | 0.00 |  | PROLACTIN | 0.00 | 0.00 | 0.00 |
| Eotaxin-3 | 38.30 | 43.75 | 41.05 |  | PSA | 85.11 | 95.83 | 90.53 |
| EPCAM | 87.23 | 91.67 | 89.47 |  | PTH | 2.13 | 0.00 | 1.05 |
| ErbB2 | 0.00 | 0.00 | 0.00 |  | RANKL | 55.32 | 58.33 | 56.84 |
| FGF-b | 14.89 | 22.92 | 18.95 |  | RANTES | 0.00 | 0.00 | 0.00 |
| Fibrinogen | 0.00 | 0.00 | 0.00 |  | Resistin | 0.00 | 0.00 | 0.00 |
| Fibronectin | 19.15 | 10.42 | 14.74 |  | SAA | 2.13 | 0.00 | 1.05 |
| Flt-3 Ligand | 25.53 | 25.00 | 25.26 |  | SAP | 0.00 | 0.00 | 0.00 |
| Fractalkine | 38.30 | 50.00 | 44.21 |  | SCC | 0.00 | 0.00 | 0.00 |
| FSH | 0.00 | 0.00 | 0.00 |  | sCD40L | 0.00 | 0.00 | 0.00 |
| G-CSF | 55.32 | 62.50 | 58.95 |  | SCF | 34.04 | 20.83 | 27.37 |
| GH | 0.00 | 0.00 | 0.00 |  | SCGF-B | 0.00 | 0.00 | 0.00 |
| GM-CSF | 78.72 | 83.33 | 81.05 |  | SDF-1a+B | 0.00 | 0.00 | 0.00 |
| GROa | 70.21 | 89.58 | 80.00 |  | sE-Selectin | 0.00 | 2.08 | 1.05 |
| HE4 | 0.00 | 0.00 | 0.00 |  | sFas | 0.00 | 0.00 | 0.00 |
| HGF | 0.00 | 0.00 | 0.00 |  | sFasL | 4.26 | 2.08 | 3.16 |
| HSA | 0.00 | 0.00 | 0.00 |  | sICAM-1 | 0.00 | 0.00 | 0.00 |
| HSP 70 | 72.34 | 81.25 | 76.84 |  | sIL-1R1 | 0.00 | 0.00 | 0.00 |
| I-309 | 10.64 | 14.58 | 12.63 |  | sIL-1RII | 0.00 | 2.08 | 1.05 |
| IFN-α | 44.68 | 77.08 | 61.05 |  | sIL-4R | 0.00 | 0.00 | 0.00 |
| IGFBP-1 | 0.00 | 0.00 | 0.00 |  | sIL-6R | 0.00 | 0.00 | 0.00 |
| IGFBP-2 | 6.38 | 2.08 | 4.21 |  | sRAGE | 4.26 | 2.08 | 3.16 |
| IGFBP-3 | 0.00 | 0.00 | 0.00 |  | sTNFRI | 0.00 | 0.00 | 0.00 |
| IGFBP-4 | 12.77 | 8.33 | 10.53 |  | sTNFRII | 0.00 | 0.00 | 0.00 |
| IGFBP-5 | 10.64 | 16.67 | 13.68 |  | sVCAM-1 | 0.00 | 0.00 | 0.00 |
| IGFBP-6 | 0.00 | 0.00 | 0.00 |  | sVEGFR2 | 0.00 | 0.00 | 0.00 |
| IGFBP-7 | 0.00 | 0.00 | 0.00 |  | sVEGFR3 | 27.66 | 25.00 | 26.32 |
| IL-11 | 0.00 | 0.00 | 0.00 |  | TARC | 0.00 | 0.00 | 0.00 |
| IL-12p70 | 34.04 | 43.75 | 38.95 |  | Tg II | 6.38 | 6.25 | 6.32 |
| IL-12p40 | 2.13 | 0.00 | 1.05 |  | TGFα | 46.81 | 54.17 | 50.53 |
| IL-15 | 10.64 | 16.67 | 13.68 |  | TSP | 0.00 | 0.00 | 0.00 |
| IL-16 | 0.00 | 0.00 | 0.00 |  | TIMP-1 | 0.00 | 0.00 | 0.00 |
| IL-17 | 97.87 | 100.00 | 98.95 |  | TIMP-2 | 0.00 | 0.00 | 0.00 |
| IL-1α | 48.94 | 70.83 | 60.00 |  | TIMP-3 | 23.40 | 20.83 | 22.11 |
| IL-1β | 21.28 | 33.33 | 27.37 |  | TIMP-4 | 0.00 | 0.00 | 0.00 |
| IL-20 | 44.68 | 54.17 | 49.47 |  | TNFα | 91.49 | 91.67 | 91.58 |
| IL-28A | 57.45 | 77.08 | 67.37 |  | TNFβ | 85.11 | 85.42 | 85.26 |
| IL-29/IFNγ | 0.00 | 0.00 | 0.00 |  | tPAI-1 | 0.00 | 0.00 | 0.00 |
| IL-3 | 80.85 | 91.67 | 86.32 |  | TPO | 0.00 | 0.00 | 0.00 |
| IL-33 | 48.94 | 66.67 | 57.89 |  | TRAIL | 2.13 | 2.08 | 2.11 |
| IL-5 | 93.62 | 100.00 | 96.84 |  | TSH | 0.00 | 0.00 | 0.00 |
| IL-7 | 36.17 | 62.50 | 49.47 |  | TSLP | 29.79 | 45.83 | 37.89 |
| IL-8 | 0.00 | 0.00 | 0.00 |  | TTR | 0.00 | 0.00 | 0.00 |
| Insulin | 19.15 | 25.00 | 22.11 |  | VEGF | 57.45 | 75.00 | 66.32 |
| Involucrin | 0.00 | 0.00 | 0.00 |  | XCL1/Lymphotactin | 14.89 | 2.08 | 8.42 |
| IP-10 | 0.00 | 0.00 | 0.00 |  |  |  |  |  |
